# Supplementary material for: Clonal dynamics after allogeneic haematopoietic cell transplantation
Source: Nature. 2024 Oct 30;635(8040):926–34. doi: 10.1038/s41586-024-08128-y (PMC11602715; doi:10.1038/s41586-024-08128-y)
Supplement: Supplementary file 1 — Supplementary Figs. 1–8 and Supplementary Tables 1 and 2. [file 41586_2024_8128_MOESM1_ESM.pdf]

nature portfolio

https://doi.org/10.1038/s41586-024-08128-y

Supplementary information

Clonal dynamics after allogeneic haematopoietic cell transplantation

In the format provided by the authors and unedited

Supplementary Information

Clonal dynamics after allogeneic haematopoietic cell transplantation

Authors: Michael Spencer Chapman[1,2,3], C. Matthias Wilk[4], Steffen Boettcher[4], Emily Mitchell[1,2,3], Kevin Dawson[1], Nicholas Williams[1], Jan Müller[4], Larisa Kovtonyuk[4], Hyunchul Jung[1], Francisco Caiado[4], Kirsty Roberts[1], Laura O'Neill[1], David G Kent[2,3,5], Anthony R. Green[2,3], Jyoti Nangalia[1,2,3], Markus G. Manz[4] *, Peter J. Campbell[1,2,3] *

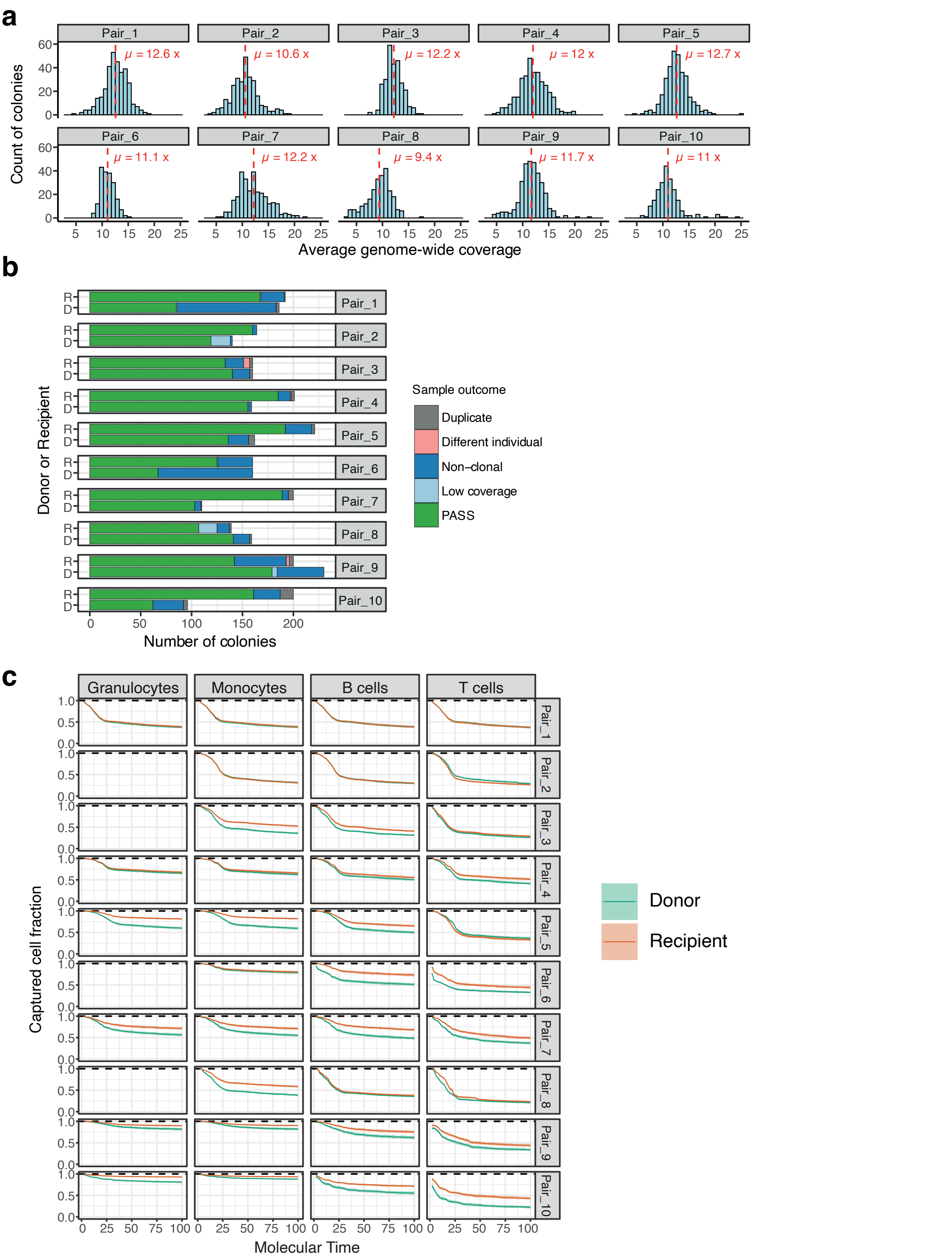

Supplementary Fig. 1 | Sequencing metrics and quality control. a, Histograms of the mean whole genome sequencing coverage of colonies used in the final data set. Red dashed lines indicate the mean value across an individual, which is also printed on each panel. **b**, Bar plot showing the outcome of all colonies undergoing WGS, divided by sibling transplant pair and donor or recipient status. **c**, Line plot showing the sum of clonal fractions across the branches of the phylogenetic tree at different points in molecular time. This is divided by pair and by cell type. The earliest time point shows the sum of clonal contributions of the first two blastomeres of the embryo. Solid line shows the median posterior values, shaded areas show the 95% posterior intervals.

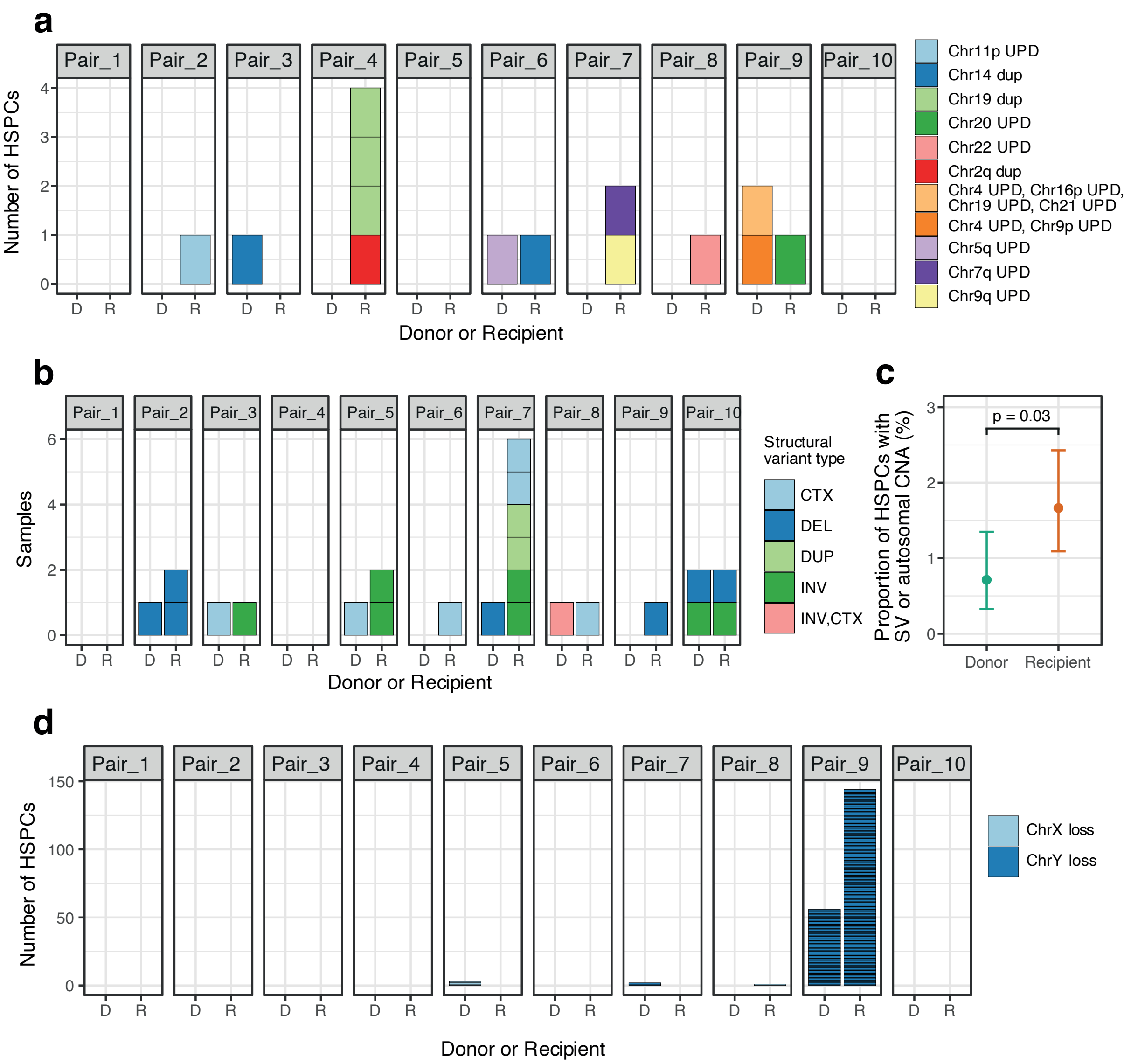

Supplementary Fig. 2 | Copy number alterations and structural variants. a, Stacked bar plot showing the number of HSPCs with autosomal CNAs in each individual, coloured by CNA type. **b,** Stacked bar plot showing the number of HSPCs with structural variants (SVs) in each individual, coloured by the class of SV. **c**, Dot plot showing the proportion of HSPCs harbouring either a CNA or SV combined across all HCT donors (green) and recipients (orange). Error bars show the 95% confidence interval (binomial test) with the p value calculated using Pearson's chi-square test. **d**, As in a, but for CNAs in the X and Y chromosomes. UPD, Uniparental disomy; dup, duplication; del, deletion; CTX, reciprocal translocation; INV, inversion: INV/ CTX, complex inversion and translocation event.

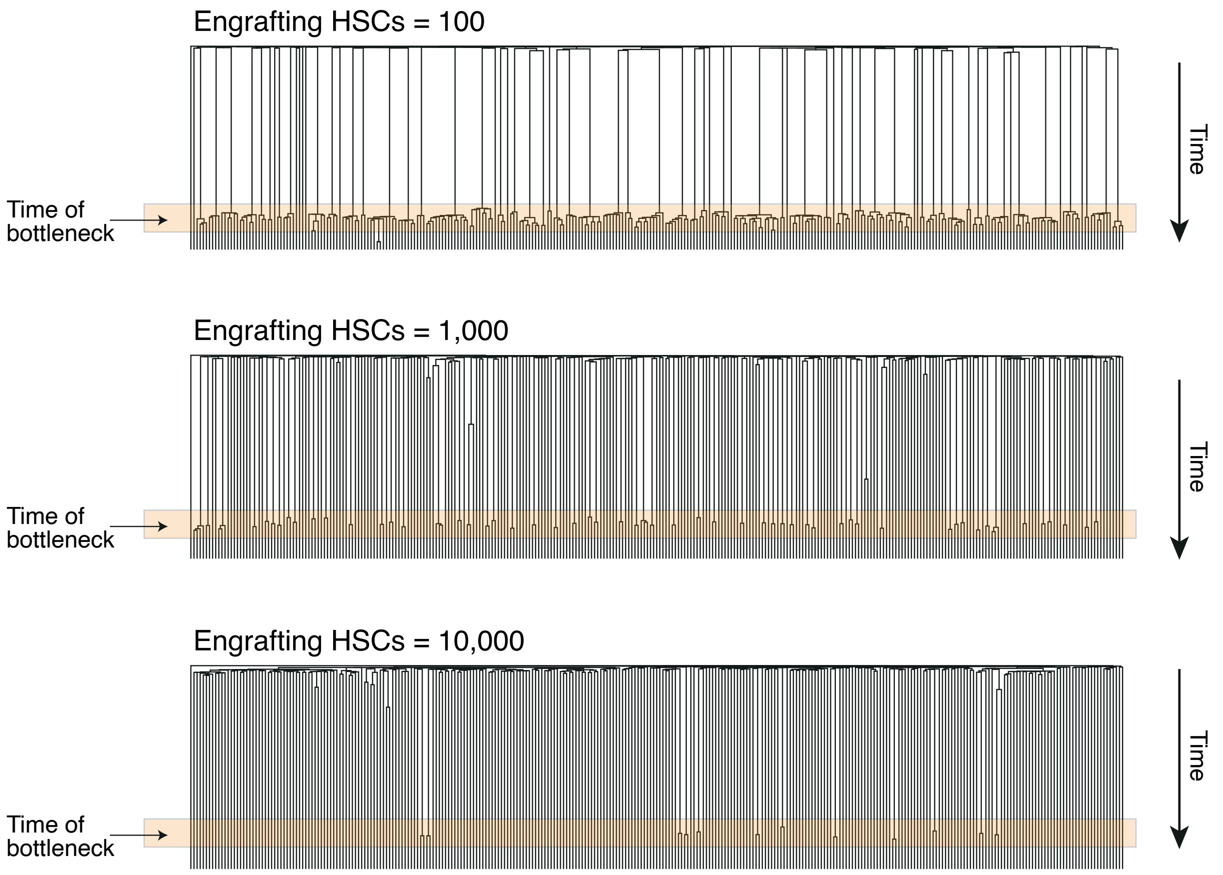

Supplementary Fig. 3 | Bottleneck simulations. Simulations of a transplant bottleneck of varying degrees. Left - bottleneck of 100 HSCs, middle - bottleneck of 1000 HSCs, right - bottleneck of 10,000 HSCs. The orange box highlights the time of the bottleneck. See methods for details of simulation framework.

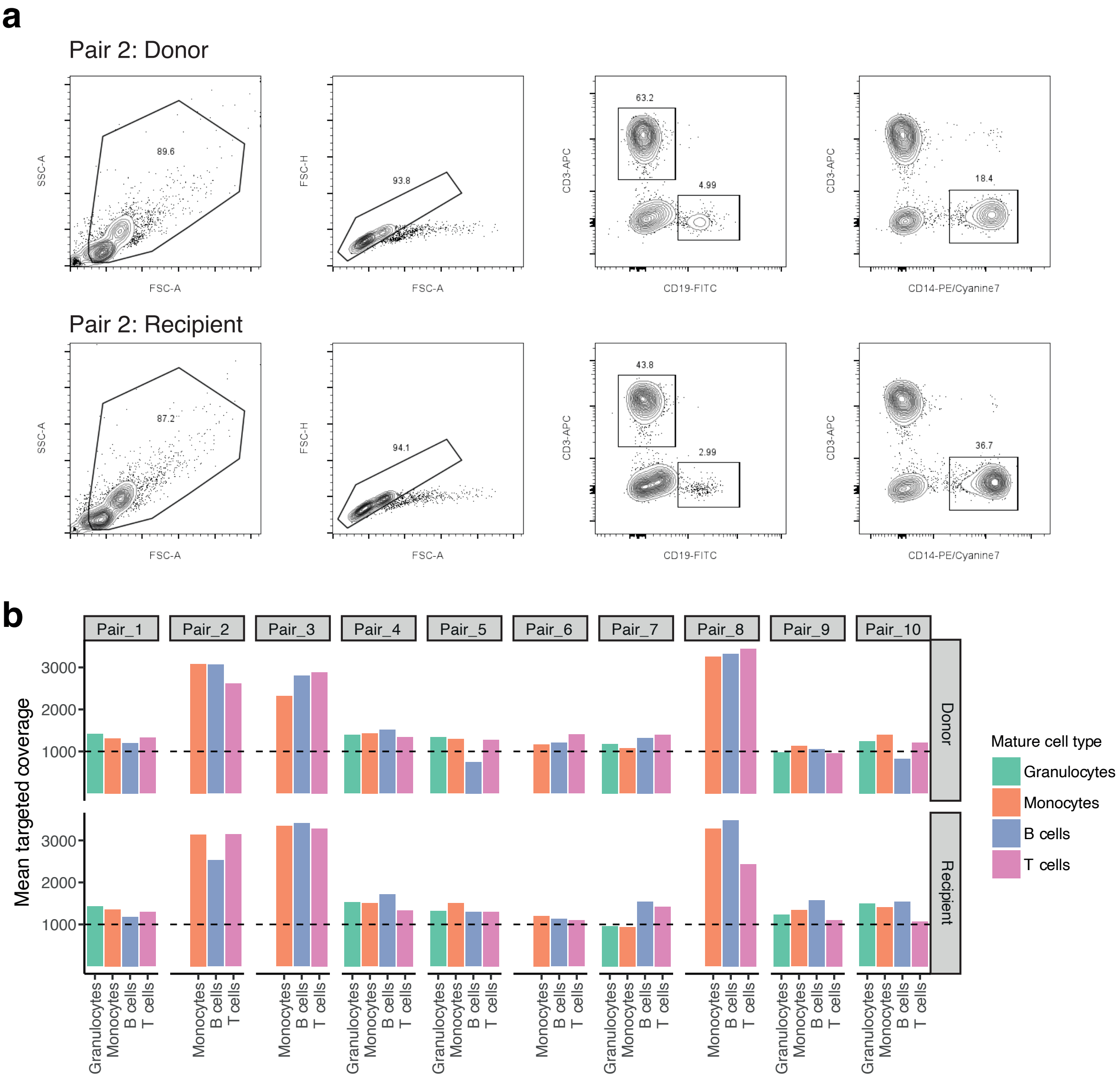

Supplementary Fig. 4 | Deep targeted sequencing of flow-sorted mature cell populations. a, Gating strategy for flow cytometric cell sorting of B-cells, T-cells and monocytes from peripheral blood cells, as illustrated by the gating used for Donor and Recipient Pair 2. **b**, Mean target sequencing coverage of bulk samples undergoing targeted sequencing, using a bait-capture approach. Results are divided by individual and cell type. Eight of 20 individuals did not have granulocytes available for sequencing. The horizontal dashed line shows the 1000x coverage value - the minimum targeted in these experiments.

a

Pair_7 combined tree: Deep targeted sequencing of granulocytes from donor

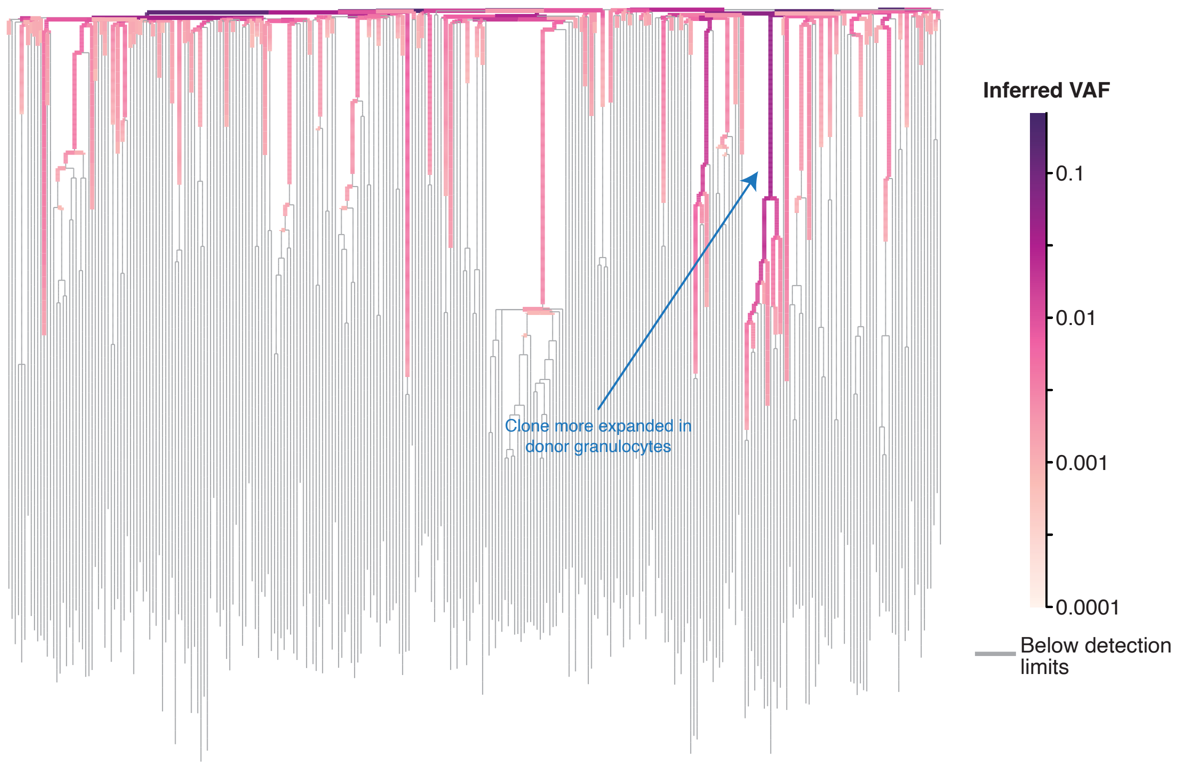

b

Pair_7 combined tree: Deep targeted sequencing of granulocytes from recipient

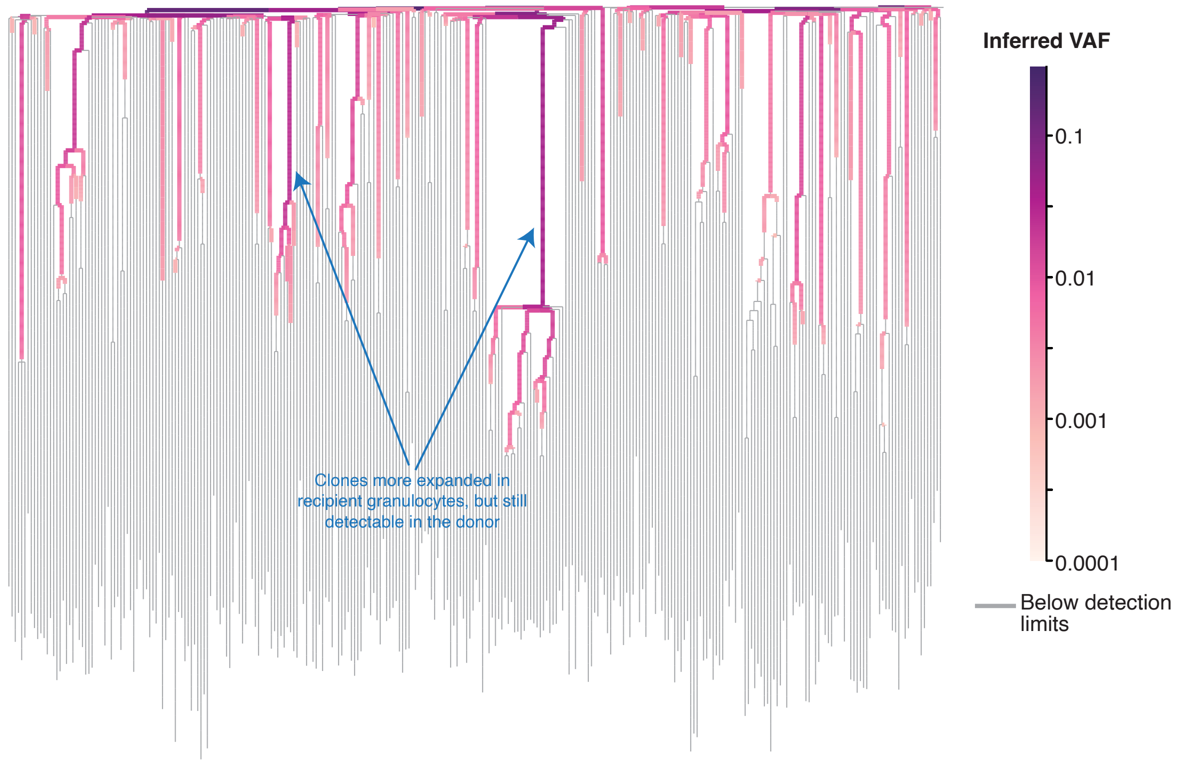

Supplementary Fig. 5 | Bayesian inference of variant allele fractions from deep targeted sequencing. a, Combined phylogeny for Pair_7, with mutations coloured by the estimated posterior median VAF from deep targeted sequencing data on a sample of granulocytes from the donor. **b**, as in **a**, but for a sample of granulocytes from the recipient.

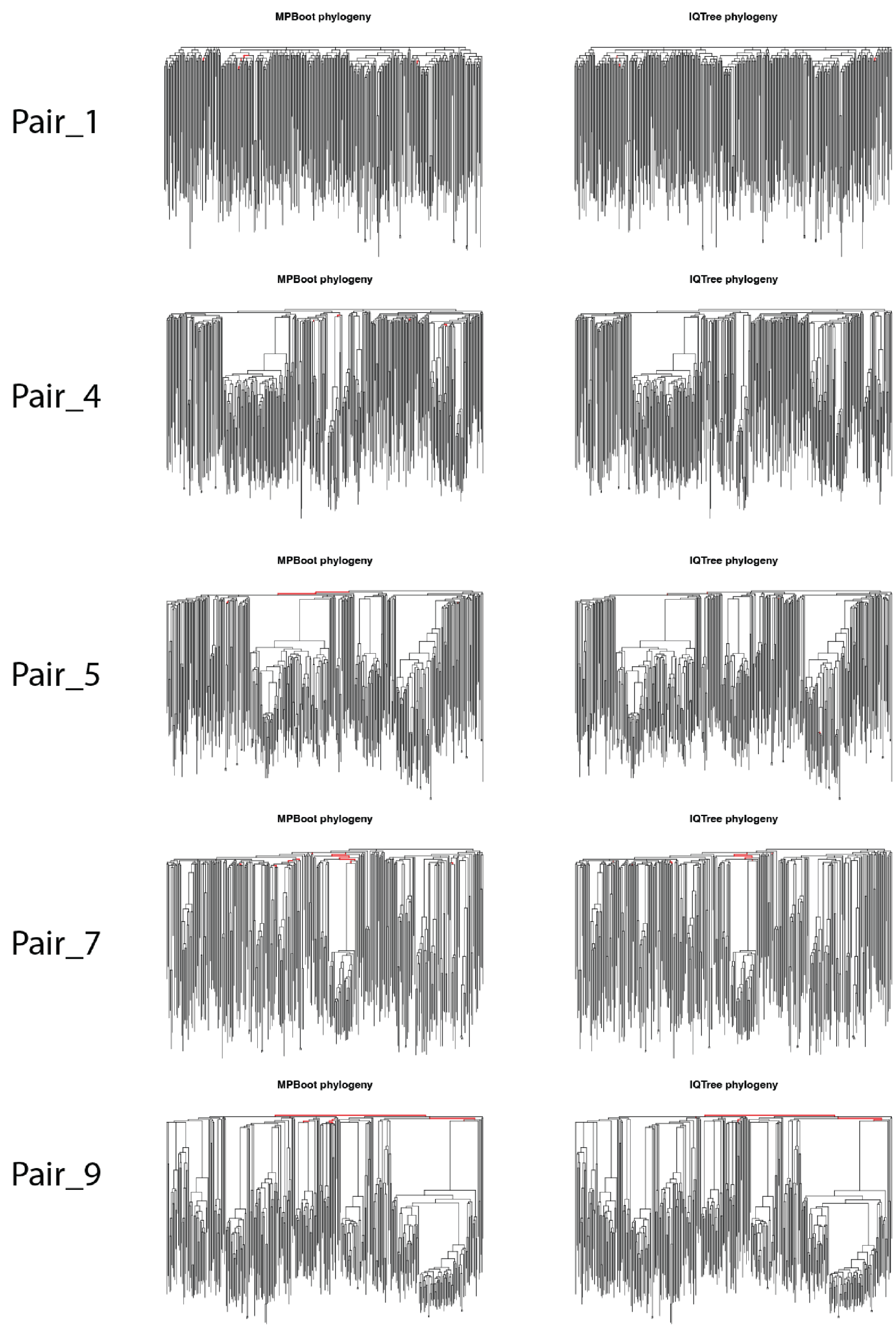

Supplementary Fig. 6 | Comparison of five phylogenies generated by MPBoot with those generated by IQtree. These are the raw phylogenies without branch length correction or removal of duplicates. Short branches with <10 mutations assigned have been extended to a length of 10 to facilitate visualisation of differences. Branches present in one tree but absent in the other are highlighted in red.

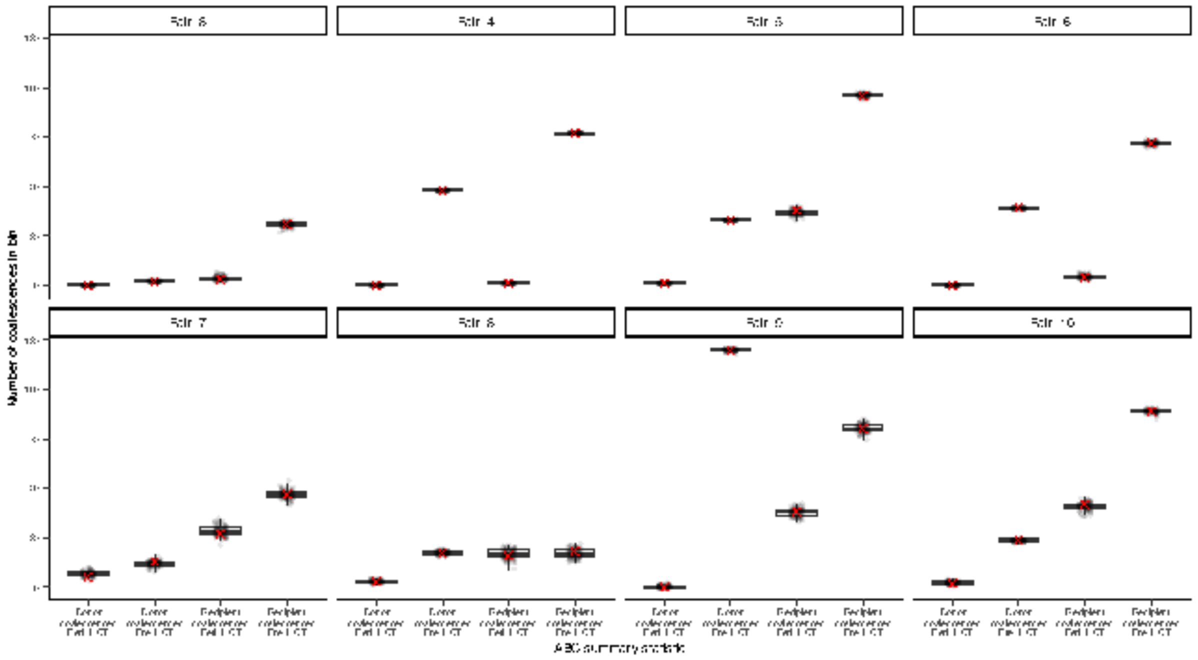

Supplementary Fig. 7 | Robustness of the coalescence-timing summary statistics to bootstrapping. For each pair, the four coalescence-timing summary statistics for the data are shown as red crosses. Those for the 100 bootstraps are shown as “box-and-whisker” plots with the raw data shown as black circles with some jittering.

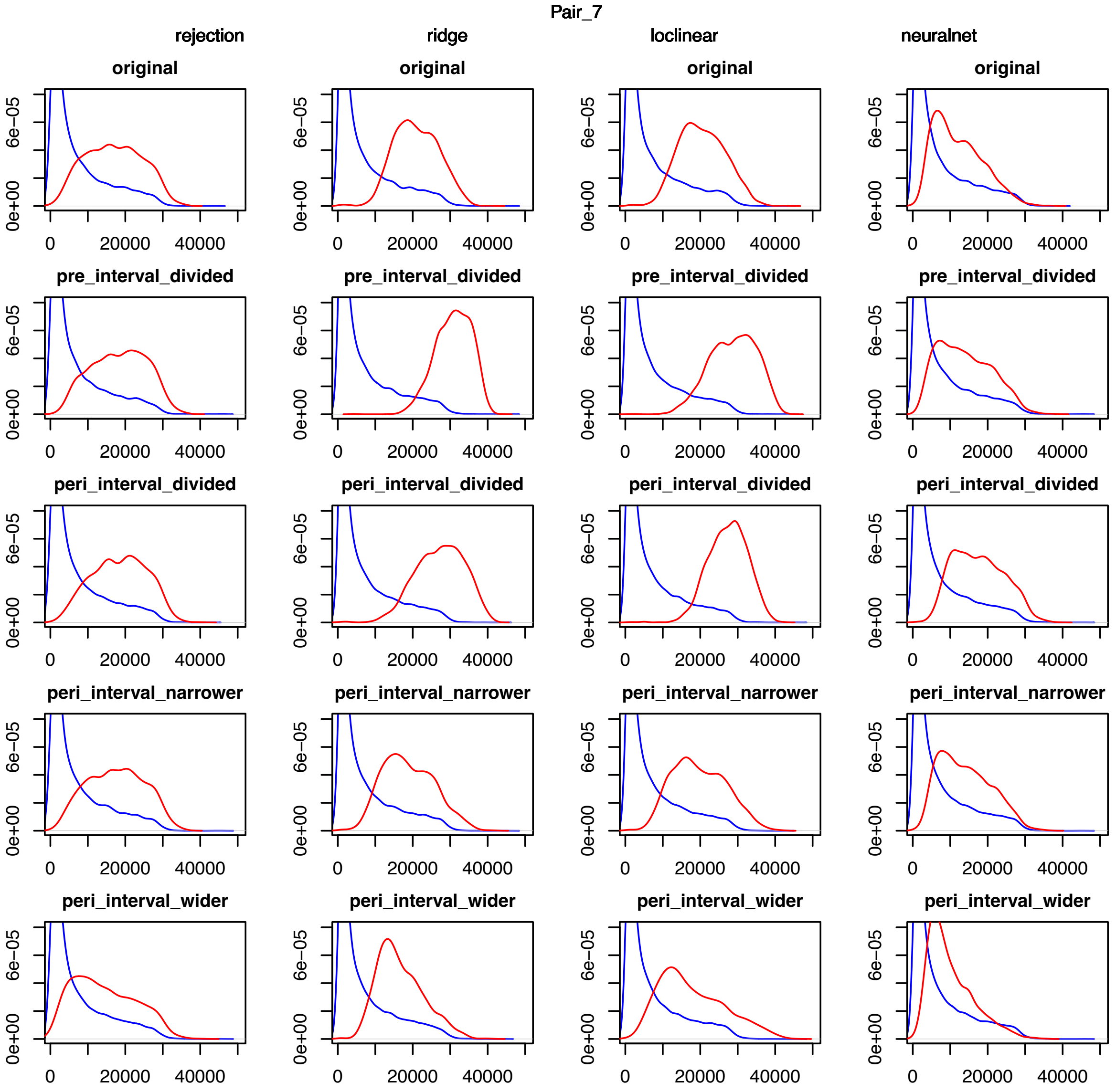

Supplementary Fig. 8 | Robustness of the posterior distributions to different definitions of epochs used in the summary statistics. Each facet plot shows the prior (blue) and estimated posterior (red) distributions for size of the HSCT bottleneck for one of the donor-recipient pairs (Pair 7). From left to right, the facets are organised by different regression methods for estimating the posterior parameter values within the ABC. From top to bottom, the facets are organised by the different definitions of epochs (original method on top followed by the four different definitions described above).

Supplementary Table 1 | List of genes assessed for possible driver mutations in haematopoietic stem cell transplant donor-recipient pairs.

ABL1	*ALK*	*ARID1A*	*ARID2*	*ASXL1*	*ASXL2*
ATRX	*BAP1*	*BAX*	*BCL10*	*BCL2*	*BCOR*
BCORL1	*BRAF*	*BRCC3*	*CALR*	*CBL*	*CBLB*
CBLC	*CCDC115*	*CCL22*	*CDK4*	*CDKN1B*	*CDKN2A*
CDKN2B	*CDKN2C*	*CEBPA*	*CHEK2*	*CREBBP*	*CRLF2*
CSF1R	*CSF3R*	*CTCF*	*CTNNB1*	*CUX1*	*DICER1*
DNMT3A	*DNMT3B*	*DUSP22*	*EED*	*EGFR*	*EP300*
ETV6	*EZH2*	*FAM175A*	*FBXW7*	*FGFR2*	*FLT3*
GATA1	*GATA2*	*GNAS*	*GNB1*	*H3F3A*	*H3F3B*
HRAS	*IDH1*	*IDH2*	*IGLL5*	*IKZF1*	*IRF4*
JAK2	*JAK3*	*KDM5C*	*KDM6A*	*KIT*	*KMT2A*
KRAS	*MAGEC3*	*MGA*	*MPL*	*MTA2*	*MYC*
MYD88	*NF1*	*NF2*	*NFE2L2*	*NOTCH1*	*NOTCH2*
NPM1	*NRAS*	*PAX5*	*PDGFRA*	*PHF6*	*PHIP*
PIK3CA	*PPM1D*	*PTEN*	*PTPN11*	*RAC1*	*RAD21*
RAD50	*RAD51*	*RB1*	*RHOA*	*RRAS*	*RUNX1*
SETBP1	*SETD2*	*SF3B1*	*SH2B3*	*SIK3*	*SMC1A*
SMC3	*SPRED2*	*SRCAP*	*SRSF1*	*SRSF2*	*STAG2*
STAT3	*STAT5A*	*SUZ12*	*TERT*	*TET2*	*TP53*
U2AF1	*WHSC1*	*WT1*	*YLPM1*	*ZBTB33*	*ZNF234*
ZNF318	*ZRSR2*				

Supplementary Table 2 | Assessment of possible driver mutations in haematopoietic stem cell transplant donor-recipient pairs. The mutation reference is given as chromosome-position-reference-variant allele. The decision column gives the assessment of whether the variant was considered a driver or variant of unknown significance (VUS).

Mutation reference	Node	Gene	Protein change	Pair	Decision
15-90633818-C-T	2	*IDH2*	IDH2 p.R89H	Pair11	VUS
16-3819192-C-T	130	*CREBBP*	CREBBP p.G1015R	Pair11	VUS
17-29557401-G-A	449	*NF1*	NF1 p.?	Pair11	VUS
17-29559129-T-C	404	*NF1*	NF1 p.L1079P	Pair11	VUS
17-7577120-C-T	16	*TP53*	TP53 p.R273H	Pair11	Driver
19-17950455-A-T	147	*JAK3*	JAK3 p.D424E	Pair11	VUS
22-41573996-A-G	174	*EP300*	EP300 p.N2094S	Pair11	VUS
2-25466800-G-A	324	*DNMT3A*	DNMT3A p.R635W	Pair11	Driver
3-105470425-G-A	63	*CBLB*	CBLB p.H202Y	Pair11	VUS
3-105572309-C-T	51	*CBLB*	CBLB p.R123K	Pair11	VUS
4-106180795-G-A	577	*TET2*	TET2 p.G1275R	Pair11	Driver
X-119387292-T-G	131	*ZBTB33*	ZBTB33 p.S8A	Pair11	VUS
X-140984370-G-A	256	*MAGEC3*	MAGEC3 p.?	Pair11	VUS
X-39913297-T-C	63	*BCOR*	BCOR p.?	Pair11	Driver
10-112361839-G-A	214	*SMC3*	SMC3 p.R1003H	Pair13	VUS
11-118374282-A-G	365	*KMT2A*	KMT2A p.T2559A	Pair13	VUS
11-32450128-G-C	115	*WT1*	WT1 p.Y228*	Pair13	Driver
13-28644741-A-G	66	*FLT3*	FLT3 p.S18P	Pair13	VUS
14-95569687-T-C	132	*DICER1*	DICER1 p.K1349R	Pair13	VUS
16-30734292-A-G	31	*SRCAP*	SRCAP p.T1301A	Pair13	VUS
16-67650648-G-A	193	*CTCF*	CTCF p.G318D	Pair13	VUS
17-58740653-C-T	542	*PPM1D*	PPM1D p.Q520*	Pair13	Driver
17-7578457-C-T	35	*TP53*	TP53 p.R158H	Pair13	Driver
2-25462020-C-T	485	*DNMT3A*	DNMT3A p.G796D	Pair13	Driver
2-25982451-T-C	266	*ASXL2*	ASXL2 p.N280S	Pair13	VUS
20-31024242-C-T	529	*ASXL1*	ASXL1 p.Q1243*	Pair13	Driver
22-23237636-G-T	165	*IGLL5*	IGLL5 p.C136F	Pair13	VUS
22-41545841-G-A	252	*EP300*	EP300 p.C819Y	Pair13	VUS
4-84406209-G-T	137	*FAM175A*	FAM175A p.T6K	Pair13	VUS
6-43322772-G-C	187	*ZNF318*	ZNF318 p.S767C	Pair13	VUS
7-101840562-G-A	76	*CUX1*	CUX1 p.R635H	Pair13	Driver
7-50468212-T-G	71	*IKZF1*	IKZF1 p.C441G	Pair13	VUS
9-133760615-C-T	17	*ABL1*	ABL1 p.P999S	Pair13	VUS
9-139402495-T-C	268	*NOTCH1*	NOTCH1 p.Y1141C	Pair13	VUS
X-119388946-T-C	234	*ZBTB33*	ZBTB33 p.F559S	Pair13	VUS
X-53441799-T-C	198	*SMC1A*	SMC1A p.I107V	Pair13	VUS
6-79655862-CG-C	487	*PHIP*	PHIP p.N1495fs*19	Pair13	Driver
1-120460347-C-T	302	*NOTCH2*	NOTCH2 p.V1990M	Pair21	VUS
1-27101603-C-T	5	*ARID1A*	ARID1A p.P1629S	Pair21	VUS
10-112352918-C-A	357	*SMC3*	SMC3 p.R634S	Pair21	VUS
11-116730042-G-T	307	*SIK3*	SIK3 p.L796I	Pair21	VUS
19-13054349-A-G	357	*CALR*	CALR p.?	Pair21	VUS
2-198273204-A-C	299	*SF3B1*	SF3B1 p.S336A	Pair21	VUS
2-25457177-G-A	566	*DNMT3A*	DNMT3A p.P904S	Pair21	Driver

**2-25458601-T-A**	381	*DNMT3A*	DNMT3A p.I858F	Pair21	Driver
**2-25459851-T-C**	196	*DNMT3A*	DNMT3A p.D811G	Pair21	Driver
**2-25463541-G-A**	509	*DNMT3A*	DNMT3A p.S714F	Pair21	Driver
**2-25466766-C-T**	59	*DNMT3A*	DNMT3A p.?	Pair21	Driver
**2-25469965-G-T**	357	*DNMT3A*	DNMT3A p.Y359*	Pair21	Driver
**2-25470497-C-G**	506	*DNMT3A*	DNMT3A p.R326P	Pair21	Driver
**22-41533746-G-A**	601	*EP300*	EP300 p.W571*	Pair21	Driver
**3-47147587-C-T**	158	*SETD2*	SETD2 p.C1580Y	Pair21	VUS
**4-106156005-T-A**	252	*TET2*	TET2 p.D302E	Pair21	Driver
**4-106164017-G-A**	269	*TET2*	TET2 p.R1176K	Pair21	Driver
**4-55129867-C-T**	173	*PDGFRA*	PDGFRA p.T134M	Pair21	VUS
**7-140453140-T-C**	65	*BRAF*	BRAF p.T599A	Pair21	Driver
**9-37014993-C-T**	243	*PAX5*	PAX5 p.?	Pair21	VUS
**X-133549118-G-A**	326	*PHF6*	PHF6 p.V268I	Pair21	Driver
**X-39933871-C-T**	528	*BCOR*	BCOR p.R243H	Pair21	Driver
**2-25463235-CAGA-C**	77	*DNMT3A*	DNMT3A p.F752delF	Pair21	Driver
**X-15841219-CG-C**	34	*ZRSR2*	ZRSR2 p.G436fs*>53	Pair21	Driver
**1-120491683-T-C**	82	*NOTCH2*	NOTCH2 p.K849R	Pair24	VUS
**10-123310935-G-A**	116	*FGFR2*	FGFR2 p.R165W	Pair24	VUS
**12-111885134-G-A**	337	*SH2B3*	SH2B3 p.G341D	Pair24	VUS
**12-12871080-G-T**	23	*CDKN1B*	CDKN1B p.A103S	Pair24	VUS
**12-58145290-C-T**	288	*CDK4*	CDK4 p.V71I	Pair24	VUS
**17-29667647-G-A**	275	*NF1*	NF1 p.R2349H	Pair24	VUS
**17-40457631-C-A**	174	*STAT5A*	STAT5A p.L462M	Pair24	VUS
**19-33793139-G-T**	6	*CEBPA*	CEBPA p.S61Y	Pair24	Driver
**2-25457242-C-T**	103	*DNMT3A*	DNMT3A p.R882H	Pair24	Driver
**2-25463248-G-T**	302	*DNMT3A*	DNMT3A p.R749S	Pair24	Driver
**2-25463291-G-C**	123	*DNMT3A*	DNMT3A p.F734L	Pair24	Driver
**2-25467478-T-C**	236	*DNMT3A*	DNMT3A p.Y533C	Pair24	Driver
**20-31016179-G-A**	258	*ASXL1*	ASXL1 p.S142N	Pair24	VUS
**20-57415846-G-A**	26	*GNAS*	GNAS p.A229T	Pair24	VUS
**22-41523536-C-T**	7	*EP300*	EP300 p.P318S	Pair24	VUS
**5-1264660-C-T**	180	*TERT*	TERT p.R901Q	Pair24	VUS
**5-1293700-G-A**	40	*TERT*	TERT p.S434F	Pair24	VUS
**6-394906-T-C**	49	*IRF4*	IRF4 p.L101S	Pair24	VUS
**X-119389023-C-T**	239	*ZBTB33*	ZBTB33 p.R585C	Pair24	Driver
**X-123197891-T-A**	208	*STAG2*	STAG2 p.F672Y	Pair24	VUS
**X-140969583-G-T**	287	*MAGEC3*	MAGEC3 p.?	Pair24	VUS
**1-36932452-G-C**	227	*CSF3R*	CSF3R p.L700V	Pair25	VUS
**1-51439893-T-A**	394	*CDKN2C*	CDKN2C p.V153D	Pair25	VUS
**11-119146845-G-A**	328	*CBL*	CBL p.?	Pair25	Driver
**11-119156151-A-G**	221	*CBL*	CBL p.S606G	Pair25	Driver
**11-62364125-T-A**	302	*MTA2*	MTA2 p.D289V	Pair25	VUS
**12-111856326-C-T**	251	*SH2B3*	SH2B3 p.P126L	Pair25	VUS
**12-111885286-C-T**	56	*SH2B3*	SH2B3 p.R392W	Pair25	Driver
**15-42046674-G-A**	213	*MGA*	MGA p.V2350M	Pair25	VUS
**16-30745085-C-T**	134	*SRCAP*	SRCAP p.R2154*	Pair25	Driver
**16-3786782-T-C**	249	*CREBBP*	CREBBP p.S1477G	Pair25	VUS
**16-57394357-G-A**	5	*CCL22*	CCL22 p.G28S	Pair25	VUS
**16-67650758-A-G**	340	*CTCF*	CTCF p.M355V	Pair25	VUS

**17-29665144-G-A**	146	*NF1*	NF1 p.R2269H	Pair25	VUS
**17-29670138-G-A**	205	*NF1*	NF1 p.G2392R	Pair25	VUS
**17-40477009-C-G**	197	*STAT3*	STAT3 p.W479S	Pair25	VUS
**2-25463295-T-G**	262	*DNMT3A*	DNMT3A p.E733A	Pair25	Driver
**2-25469633-G-A**	140	*DNMT3A*	DNMT3A p.R379C	Pair25	Driver
**2-25470021-C-T**	96	*DNMT3A*	DNMT3A p.V341I	Pair25	Driver
**2-65571975-C-T**	162	*SPRED2*	SPRED2 p.G28R	Pair25	VUS
**22-41527481-C-T**	335	*EP300*	EP300 p.Q458*	Pair25	Possible
**22-41573825-G-A**	197	*EP300*	EP300 p.G2037D	Pair25	VUS
**3-41278186-A-G**	59	*CTNNB1*	CTNNB1 p.M688V	Pair25	VUS
**3-47127765-G-A**	271	*SETD2*	SETD2 p.R1773C	Pair25	VUS
**3-47162156-G-A**	363	*SETD2*	SETD2 p.Q1324*	Pair25	Driver
**4-106155386-G-A**	522	*TET2*	TET2 p.R96H	Pair25	Driver
**4-153332496-C-T**	57	*FBXW7*	FBXW7 p.V154I	Pair25	VUS
**6-401645-G-A**	266	*IRF4*	IRF4 p.G323R	Pair25	VUS
**9-139404329-C-T**	225	*NOTCH1*	NOTCH1 p.C942Y	Pair25	VUS
**1-27087485-C-T**	201	*ARID1A*	ARID1A p.P687S	Pair28	VUS
**12-46245036-C-T**	446	*ARID2*	ARID2 p.Q1044*	Pair28	Driver
**2-25457192-G-C**	249	*DNMT3A*	DNMT3A p.R899G	Pair28	Driver
**21-36421183-C-T**	237	*RUNX1*	RUNX1 p.S5N	Pair28	VUS
**22-29083956-G-A**	429	*CHEK2*	CHEK2 p.R564W	Pair28	Driver
**22-29090060-C-T**	414	*CHEK2*	CHEK2 p.R517H	Pair28	Driver
**3-41266573-C-A**	202	*CTNNB1*	CTNNB1 p.R124S	Pair28	VUS
**3-47147564-C-T**	201	*SETD2*	SETD2 p.E1588K	Pair28	VUS
**3-47162848-C-T**	28	*SETD2*	SETD2 p.S1093N	Pair28	VUS
**4-106190818-C-T**	215	*TET2*	TET2 p.R1366C	Pair28	Driver
**4-106158085-GC-G**	252	*TET2*	TET2 p.A996fs*11	Pair28	Driver
**X-154348284-GA-G**	261	*BRCC3*	BRCC3 p.N271fs*45	Pair28	Driver
**1-120497790-T-C**	131	*NOTCH2*	NOTCH2 p.I698V	Pair31	VUS
**1-120512209-C-T**	125	*NOTCH2*	NOTCH2 p.A345T	Pair31	VUS
**11-118374730-A-C**	119	*KMT2A*	KMT2A p.E2708A	Pair31	VUS
**14-95574698-C-T**	8	*DICER1*	DICER1 p.R800K	Pair31	VUS
**16-30735573-C-T**	146	*SRCAP*	SRCAP p.P1610S	Pair31	VUS
**16-3823897-G-A**	175	*CREBBP*	CREBBP p.P773L	Pair31	VUS
**17-29548893-A-G**	151	*NF1*	NF1 p.D556G	Pair31	VUS
**17-58740550-G-T**	27	*PPM1D*	PPM1D p.R485S	Pair31	Driver
**19-44662159-C-T**	60	*ZNF234*	ZNF234 p.R664*	Pair31	Driver
**2-25505420-C-T**	41	*DNMT3A*	DNMT3A p.G113E	Pair31	Driver
**2-25994403-G-A**	25	*ASXL2*	ASXL2 p.S137L	Pair31	VUS
**X-39932819-C-T**	86	*BCOR*	BCOR p.V594I	Pair31	Driver
**22-30061020-GT-G**	13	*NF2*	NF2 p.F285fs*11	Pair31	Driver
**15-42032261-A-G**	106	*MGA*	MGA p.N1482S	Pair38	VUS
**17-7577124-C-T**	366	*TP53*	TP53 p.V272M	Pair38	Driver
**2-25468888-C-A**	394	*DNMT3A*	DNMT3A p.?	Pair38	Driver
**2-25470484-C-T**	300	*DNMT3A*	DNMT3A p.W330*	Pair38	Driver
**2-25470516-G-A**	272	*DNMT3A*	DNMT3A p.R320*	Pair38	Driver
**2-29455283-A-G**	201	*ALK*	ALK p.I840T	Pair38	VUS
**20-31390276-G-A**	443	*DNMT3B*	DNMT3B p.R744K	Pair38	VUS
**20-31390276-G-C**	163	*DNMT3B*	DNMT3B p.R744T	Pair38	VUS
**3-105470368-C-A**	167	*CBLB*	CBLB p.D221Y	Pair38	VUS

**4-106180807-G-T**	149	*TET2*	TET2 p.E1279*	Pair38	Driver
**6-43316334-T-C**	206	*ZNF318*	ZNF318 p.K934E	Pair38	Driver
**X-39933205-A-G**	42	*BCOR*	BCOR p.V465A	Pair38	Driver
**1-36933786-A-G**	457	*CSF3R*	CSF3R p.I538T	Pair40	VUS
**11-119145612-C-T**	130	*CBL*	CBL p.T273M	Pair40	Driver
**11-85975280-G-T**	115	*EED*	EED p.G234V	Pair40	VUS
**16-30727487-A-G**	57	*SRCAP*	SRCAP p.Q865R	Pair40	VUS
**17-29663364-T-C**	16	*NF1*	NF1 p.L2007P	Pair40	VUS
**17-7577566-T-C**	36	*TP53*	TP53 p.N239D	Pair40	Driver
**2-25457231-G-C**	253	*DNMT3A*	DNMT3A p.Q886E	Pair40	Driver
**2-65571995-A-T**	26	*SPRED2*	SPRED2 p.M21K	Pair40	VUS
**20-57428516-G-A**	278	*GNAS*	GNAS p.E66K	Pair40	VUS
**22-29091842-G-A**	72	*CHEK2*	CHEK2 p.S415F	Pair40	Driver
**22-29099506-T-C**	355	*CHEK2*	CHEK2 p.I342V	Pair40	Driver
**5-149460421-G-T**	88	*CSF1R*	CSF1R p.N72K	Pair40	VUS
**6-43305603-C-T**	125	*ZNF318*	ZNF318 p.E2045K	Pair40	VUS
**6-43333180-T-C**	126	*ZNF318*	ZNF318 p.?	Pair40	Driver
**7-148524346-C-T**	69	*EZH2*	EZH2 p.R213H	Pair40	VUS
**X-76938554-C-A**	48	*ATRX*	ATRX p.D732Y	Pair40	VUS
**4-106156053-AC-A**	202	*TET2*	TET2 p.P319fs*28	Pair40	Driver
**13-48951138-G-A**	109	*RB1*	RB1 p.V434M	Pair41	VUS
**16-30718975-G-A**	259	*SRCAP*	SRCAP p.R192H	Pair41	Driver
**2-26068405-T-C**	18	*ASXL2*	ASXL2 p.M29V	Pair41	VUS
**4-106197336-A-G**	226	*TET2*	TET2 p.N1890S	Pair41	Driver
**4-1919899-C-T**	198	*WHSC1*	WHSC1 p.A320V	Pair41	VUS
**4-1980493-G-A**	26	*WHSC1*	WHSC1 p.G1319R	Pair41	VUS
**5-131931498-A-G**	195	*RAD50*	RAD50 p.M735V	Pair41	VUS
**9-37020654-C-A**	162	*PAX5*	PAX5 p.C64F	Pair41	VUS
**X-53423265-A-G**	64	*SMC1A*	SMC1A p.I915T	Pair41	VUS
**6-43310585-AG-A**	175	*ZNF318*	ZNF318 p.T1035fs*80	Pair41	Driver
