# Supplementary material for: Clonal dynamics after allogeneic haematopoietic cell transplantation
Source: Nature. 2024 Oct 30;635(8040):926–34. doi: 10.1038/s41586-024-08128-y (PMC11602715; doi:10.1038/s41586-024-08128-y)
Supplement: Supplementary file 2 — Reporting Summary [file 41586_2024_8128_MOESM2_ESM.pdf]

Corresponding author(s): Peter Campbell
Markus Manz

Last updated by author(s): Sep 16, 2024

Reporting Summary

Nature Portfolio wishes to improve the reproducibility of the work that we publish. This form provides structure for consistency and transparency in reporting. For further information on Nature Portfolio policies, see our Editorial Policies and the Editorial Policy Checklist.

Statistics

For all statistical analyses, confirm that the following items are present in the figure legend, table legend, main text, or Methods section.

n/a	Confirmed	
☐	☒	The exact sample size (*n*) for each experimental group/condition, given as a discrete number and unit of measurement
☐	☒	A statement on whether measurements were taken from distinct samples or whether the same sample was measured repeatedly
☐	☒	The statistical test(s) used AND whether they are one- or two-sided<br>*Only common tests should be described solely by name; describe more complex techniques in the Methods section.*
☐	☒	A description of all covariates tested
☐	☒	A description of any assumptions or corrections, such as tests of normality and adjustment for multiple comparisons
☐	☒	A full description of the statistical parameters including central tendency (e.g. means) or other basic estimates (e.g. regression coefficient) AND variation (e.g. standard deviation) or associated estimates of uncertainty (e.g. confidence intervals)
☐	☒	For null hypothesis testing, the test statistic (e.g. *F*, *t*, *r*) with confidence intervals, effect sizes, degrees of freedom and *P* value noted<br>*Give P values as exact values whenever suitable.*
☐	☒	For Bayesian analysis, information on the choice of priors and Markov chain Monte Carlo settings
☐	☒	For hierarchical and complex designs, identification of the appropriate level for tests and full reporting of outcomes
☒	☐	Estimates of effect sizes (e.g. Cohen's *d*, Pearson's *r*), indicating how they were calculated

Our web collection on statistics for biologists contains articles on many of the points above.

Software and code

Policy information about availability of computer code

Data collection	None
Data analysis	List of programs and softwares:<br>• R: version 4.1.1<br>• BWA-MEM: version 0.7.17 (https://sourceforge.net/projects/bio-bwa/)<br>• cgpCaVEMan: version 1.11.2/1.13.14/1.14.1 (https://github.com/cancerit/CaVEMan)<br>• cgpPindel: version 2.2.5/3.2.0/3.3.0 (https://github.com/cancerit/cgpPindel)<br>• ASCAT NGS: version 4.2.1/4.3.3 (https://github.com/cancerit/ascatNgs)<br>• VAGrENT: version 3.5.2/3.6.0/3.6.1 (https://github.com/cancerit/VAGrENT)<br>• GRIDSS: version 2.9.4 (https://github.com/PapenfussLab/gridss)<br>• MPBoot: version 1.1.0 (https://github.com/diepthihoang/mpboot)<br>• cgpVAF: version 2.4.0 (https://github.com/cancerit/vafCorrect)<br>• dNdScv: version 0.0.1.0 (https://github.com/im3sanger/dndscv)<br>• Rsimpop: version 2.2.6 (https://github.com/NickWilliamsSanger/rsimpop)<br>Custom code made available (also stated in manuscript): https://github.com/mspencerchapman/Clonal_dynamics_of_HSCT<br>No commercial software used.

For manuscripts utilizing custom algorithms or software that are central to the research but not yet described in published literature, software must be made available to editors and reviewers. We strongly encourage code deposition in a community repository (e.g. GitHub). See the Nature Portfolio guidelines for submitting code & software for further information.

Data

Policy information about availability of data

All manuscripts must include a data availability statement. This statement should provide the following information, where applicable:

- Accession codes, unique identifiers, or web links for publicly available datasets
- A description of any restrictions on data availability
- For clinical datasets or third party data, please ensure that the statement adheres to our policy

Whole genomes and targeted sequencing data have been deposited in the European Genome–phenome Archive (EGA) (https://ega-archive.org/). WGS data have been deposited with EGA accession number EGAD00001010872 and targeted sequencing data have been deposited with accession number EGAD00001010874. Larger files of data necessary to reproduce some of the analysis in the github repository are available on Mendeley Data (https://data.mendeley.com/datasets/m7nz2jk8wb/1).

Field-specific reporting

Please select the one below that is the best fit for your research. If you are not sure, read the appropriate sections before making your selection.

☒ Life sciences ☐ Behavioural & social sciences ☐ Ecological, evolutionary & environmental sciences

For a reference copy of the document with all sections, see nature.com/documents/nr-reporting-summary-flat.pdf

Life sciences study design

All studies must disclose on these points even when the disclosure is negative.

Sample size	We optimised the number of transplant pairs (10 pairs, 20 individuals) and number of haematopoietic stem cells sequenced per individual (average of 170 cells per individual) to describe the transplanted cell numbers, mutation burden, and clonal structure, across a range of transplant variables. No power calculation was performed, and there was no target effect size. This was an observational study.
Data exclusions	Genomes with a sequencing depth of less than 4x (46 samples), a VAF distribution showing evidence of non-clonality or contamination (peak VAF < 40%) (468 samples), or with evidence that they were from a different germline (10 samples) were excluded from the analysis. These data exclusions were made to maintain quality of mutation calls and phylogenetic inference.
Replication	While the specific donor samples used have been exhausted, the results from this study should be generally reproducible in separate transplant pairs with similar characteristics, using the protocols and code included in this manuscript.
Randomization	This is not relevant to our study because it is an observational, descriptive study. Transplant pairs were selected.
Blinding	Blinding was not relevant to our study because outcome variables were computationally determined. There was no test performed that required blinding.

Reporting for specific materials, systems and methods

We require information from authors about some types of materials, experimental systems and methods used in many studies. Here, indicate whether each material, system or method listed is relevant to your study. If you are not sure if a list item applies to your research, read the appropriate section before selecting a response.

Materials & experimental systems

n/a	Involved in the study	
☐	☒	Antibodies
☒	☐	Eukaryotic cell lines
☒	☐	Palaeontology and archaeology
☒	☐	Animals and other organisms
☐	☒	Human research participants
☒	☐	Clinical data
☒	☐	Dual use research of concern

Methods

n/a	Involved in the study	
☒	☐	ChIP-seq
☐	☒	Flow cytometry
☒	☐	MRI-based neuroimaging

Antibodies

Antibodies used	PE/Cyanine7 anti-human CD14 BioLegend #301814<br>APC anti-human CD3 BioLegend #317318<br>FITC anti-human CD19 BioLegend #363008

Validation | These were all previously validated commercially available antibodies.
CD3 FITC: Validated by supplier with the following notes - species reactivity: human; application - flow cytometry
CD19 A700: Validated by the supplier with the following notes - species reactivity: human, chimpanzee, rhesus; application: flow cytometry

Human research participants

Policy information about studies involving human research participants

Population characteristics | The dataset comprised 3,399 whole genomes from the blood of 10 fully HLA-matched sibling donor and recipient pairs (20 individuals) who had been recruited for a previous study. In each case, the recipient had undergone HCT many years prior to sampling (range: 9-31 years) and had complete or almost complete replacement of their haematopoietic system with that of the donor. The most common indication for HCT was acute myeloid leukaemia; the conditioning regimen was myelo-ablative (n=7) or reduced intensity (n=3); the stem cell source was bone marrow (n=5) or mobilised peripheral blood (n=5); and recipients were of similar age to their sibling donors (age difference: -7 to +11 years). The youngest individual was 34 years old at the time of sampling; the oldest was 79 years old. There were 13 females and 7 males in the study.

Recruitment | Individuals were recruited in Zurich, Switzerland, and ethical approval was by the local ethics board (Kantonale Ethikkommission - Zurich).
Donor and recipient pairs were recruited if both were alive at least 10 years after transplant. This biases for transplant procedures that have resulted in long-term disease remission for the recipient. This may bias against recipients with disease at high risk of relapse, or high transplant-related mortality.

Ethics oversight | Kantonale Ethikkommission - Zurich (KEK-ZH No. 2015-0053 & 2019-02290)

Note that full information on the approval of the study protocol must also be provided in the manuscript.

Flow Cytometry

Plots

Confirm that:

☒ The axis labels state the marker and fluorochrome used (e.g. CD4-FITC).

☒ The axis scales are clearly visible. Include numbers along axes only for bottom left plot of group (a 'group' is an analysis of identical markers).

☒ All plots are contour plots with outliers or pseudocolor plots.

☒ A numerical value for number of cells or percentage (with statistics) is provided.

Methodology

Sample preparation | Granulocytes were isolated from 10ml of EDTA anticoagulated peripheral blood using EasySep Direct Neutrophil Isolation Kit (StemCellTechnologies, Vancouver, Canada) according to the manufacturer’s instructions. CD34+ HSPCs were isolated from 20ml of EDTA anticoagulated peripheral blood using human CD34 MicroBead Kit (Miltenyi Biotec, Bergisch Gladbach, Germany) following the manufacturer’s recommendations. B cells, T cells and monocytes were flow-sorted from CD34- cell fractions using a FACSAria III flow cytometer (BD Biosciences, San Jose, USA).

Instrument | FACSAria II flow cytometer (BD Biosciences, San Jose, USA).

Software | No analysis of flow cytometry data is presented in this manuscript. FlowJo v10 (BD Biosciences, San Jose, USA) was used to generate the gating strategy image.

Cell population abundance | Sorting of CD3+ T cells, CD19+ B cells and CD14+ monocytes was performed on a FACSAria III flow cytometer (BD Biosciences, San Jose, USA) with resulting post-sort cell populations of >90% purity as determined by flow cytometry on the same instrument.

Gating strategy | After gating for cellular events in the FSC-A and SSC-A and doublet exclusion in the FSC-A and FSC-H plots; CD3, CD19 and CD14 were used to gate for T cells, B cells and monocytes, respectively.

☒ Tick this box to confirm that a figure exemplifying the gating strategy is provided in the Supplementary Information.
